# Targeting of CD163^+^ Macrophages in Inflammatory and Malignant Diseases

**DOI:** 10.3390/ijms21155497

**Published:** 2020-07-31

**Authors:** Maria K. Skytthe, Jonas Heilskov Graversen, Søren K. Moestrup

**Affiliations:** 1 Department of Molecular Medicine, University of Southern Denmark, 5000 Odense, Denmark; mskytthe@health.sdu.dk (M.K.S.); smoestrup@health.sdu.dk (S.K.M.); 2 Department of Biomedicine, Aarhus University, 8200 Aarhus, Denmark

**Keywords:** macrophage, CD163, inflammation, cancer, antibody-drug conjugate, targeting, glucocorticoid

## Abstract

The macrophage is a key cell in the pro- and anti-inflammatory response including that of the inflammatory microenvironment of malignant tumors. Much current drug development in chronic inflammatory diseases and cancer therefore focuses on the macrophage as a target for immunotherapy. However, this strategy is complicated by the pleiotropic phenotype of the macrophage that is highly responsive to its microenvironment. The plasticity leads to numerous types of macrophages with rather different and, to some extent, opposing functionalities, as evident by the existence of macrophages with either stimulating or down-regulating effect on inflammation and tumor growth. The phenotypes are characterized by different surface markers and the present review describes recent progress in drug-targeting of the surface marker CD163 expressed in a subpopulation of macrophages. CD163 is an abundant endocytic receptor for multiple ligands, quantitatively important being the haptoglobin-hemoglobin complex. The microenvironment of inflammation and tumorigenesis is particular rich in CD163^+^ macrophages. The use of antibodies for directing anti-inflammatory (e.g., glucocorticoids) or tumoricidal (e.g., doxorubicin) drugs to CD163^+^ macrophages in animal models of inflammation and cancer has demonstrated a high efficacy of the conjugate drugs. This macrophage-targeting approach has a low toxicity profile that may highly improve the therapeutic window of many current drugs and drug candidates.

## 1. Introduction

Macrophages are heterogenic phagocytic cells of the innate immune system with outstanding functional plasticity beyond innate immunity. For instance, macrophages are essential for maintenance of homeostasis, organ morphogenesis, tissue remodeling and repair and regulation of inflammation [1,2,3,4,5]. They are found in virtually all organs and tissues either as differentiated macrophages recruited from blood monocytes or as tissue resident macrophages that originate from the embryonic yolk sac, fetal liver or bone marrow monocytes [6]. The macrophage heterogeneity and plasticity is evident from how the microenvironment shapes macrophage phenotype and functional identity which ensures ongoing adaption of macrophages to the environment [7,8]. However, our knowledge about the functional significance of macrophage plasticity is incomplete and more evidence is needed within this field [9]. In the present review focusing on the therapeutic properties of using CD163 as a target, we introduce with short overviews on macrophage differentiation, the role of macrophages in disease, a status of macrophage targeting and the function of CD163 in healthy and diseased tissues.

## 2. Macrophage Differentiation

Macrophage activation was first introduced in 1962 by Mackaness. He observed acquired resistance to *Listeria monocytogenes* infections in mice as a result of macrophage changes [10]. It was later observed that macrophages are activated differently in response to various stimuli. First, interferon-gamma (IFN-γ) was found to stimulate macrophages towards a pro-inflammatory response with macrophage excretion of pro-inflammatory markers, a high level of antigen presentation and bactericidal and tumoricidal activity [3,11,12,13]. Later an alternative activated macrophage phenotype was described which encompasses macrophages that are not stimulated by IFN-γ [14,15]. Instead this response was stimulated by IL-4 [14], IL-13 [16], glucocorticoids [17], transforming growth factor β (TGF-β), immune complexes and IL-6 [2,18]. This stimulus results in an opposite inflammatory activation with production of anti-inflammatory responses and expression of anti-inflammatory markers. In addition, this type of macrophages is associated with tissue repair, efferocytosis, endocrine signaling, angiogenesis, tumor growth and metastasis [2,13].

Mills and colleagues introduced the M1/M2 terminology in 2000 [19], where M1s are the classically activated pro-inflammatory macrophages and M2s are the alternatively activated anti-inflammatory macrophages. They studied the L-arginine metabolism in macrophages which revealed a dichotomy corresponding to the one found for T helper cells, as two competitive metabolic states were demonstrated in murine macrophages upon either Th1 or Th2 stimuli [7,8]. Th1-stimulated macrophages (INF-γ and/or lipopolysaccharide (LPS)) resulted in upregulation of nitric oxide synthase (iNOS) which oxidizes L-arginine to nitric oxide and L-citrulline. On the other hand, arginase-1 was upregulated in Th2 stimulated macrophages (IL-4 or IL-13) thereby metabolizing L-arginine to L-ornithine and urea. However, other M2 stimulants such as IL-10, TGF-β and glucocorticoids did not fit this dichotomy, as they do not correspond to a Th2 response. Therefore, the M2 macrophages were further categorized into M2a induced by IL-4 and IL-13, M2b induced by immunocomplexes and toll-like receptor (TLR) ligands or IL-1R, M2c induced by IL-10 and glucocorticoids [5] and M2d induced by IL-6 [9] and adenosines [10]. 

However, the M1/M2 paradigm has been inadequate to explain macrophage plasticity in many studies [3,6,20,21]. For instance, macrophages are able to adopt intermediate phenotypes that present mixed M1 and M2 characteristics [22,23] and to change phenotype in response to the microenvironment [24,25,26]. Furthermore, they are stimulated by a vast number of other molecules that are linked to chronic inflammation rather than the acute inflammatory signals included in the M1/M2 paradigm [20]. Using transcriptomics, Xue et al. [20] demonstrated that human monocyte-derived macrophages polarize to a spectrum of macrophages upon activation by 28 different stimuli. A bipolar activation was achieved when stimulating with defined M1 and M2 stimuli, however, when stimulating with fatty acids, high-density lipoprotein or combinations of stimuli associated with chronic inflammation (such as the combination of tumor necrosis factor alpha (TNF-α), prostaglandin E_2_ and P3C in chronic granulomatous inflammation) a spectrum of macrophage-activation signatures appeared [20]. Thus, the two states appear as two opposite extremes with a large spectrum of macrophages in between. Mills and colleagues originally outlined this possibility when they proposed the M1/M2 terminology, however, the simplicity of the concept has been taken out of context [3]. Although the dichotomic terminology is inadequate in complex compartments in vivo and its use has been strongly debated and criticized [3,6,20,21], the nomenclature has been helpful to understand and explain the complex functions and characteristics of macrophages in the pro- and anti-inflammatory response. Most likely, an infinite number of different macrophage phenotypes exist and any future improved nomenclature may only approximate the true spectrum of macrophages. 

In addition to the inadequate description of differentiation of monocytes to the heterogenic macrophage populations in vivo [27] interspecies differences [28] and lack of conserved surface markers between the species hampers translatability of animal studies to human settings [29,30]. For instance, only murine macrophages express the highly used macrophage antigen F4/80, instead the human homolog EMR1 is predominantly expressed by eosinophilic granulocytes [31]. Also, when using the M1/M2 dichotomy, identifying M1 and M2 in mice CXCL9, CXCL10, CXCL11, NOS2 and Mrc1(CD206), tgm2, Fizz1, Ym1/2, Arg1 have been used, respectively. However, in humans CD64, IDO, SOCS1, CXCL10 have been used as M1 markers, while MRC1, TGM2, CD23, CCL22 are M2 markers [3]. Thus, only CXCL10 and transglutaminase 2 (TGM2) [29] are conserved among human and mouse representing classical and alternative activated macrophages, respectively. In addition, human CD14^++^CD16^−^ and CD16^+^ peripheral blood monocytes are transcriptionally homologous to mouse Ly6C^hi^CX3CR1^lo^ and Ly6C^lo^CX3CR1^hi^ macrophages, respectively [30]. Advances in single-cell RNA sequencing have allowed for more precise comparative analysis of mononuclear cells among species [30,32]. For instance, Zimmerman et al. [33] identified four conserved genes (*C1qc, cd74, cd81* and *Apoe*) describing renal resident macrophages across species including mouse, rat, pig and human kidney tissue using single-cell RNA sequencing. Further, using flow cytometry they demonstrated that the cell surface markers CD74 and CD81 distinguished renal resident macrophages from infiltrating macrophages in mouse, rat and human kidney tissue [33]. In another study, species-specific patterns were observed investigating tumor-infiltrating macrophages of non-small-cell lung cancer by single-cell RNA sequencing, while dendritic cells and monocytes were conserved between mouse and man [34]. More comparative studies using single-cell techniques will hopefully contribute to the knowledge of macrophage similarities and differences among species in health and disease improving the transferability of animal studies. Further, the single-cell techniques can contribute to uncover the heterogeneity of activated macrophages and help elucidate their plasticity and function over time in health and disease.

## 3. Macrophages in Disease

In response to infection and tissue injury, macrophages orchestrate a pro-inflammatory response in the early stage by recruitment, proliferation and activation of hematopoietic and non-hematopoietic cells. Later, the macrophages redirect their functional phenotype and direct an anti-inflammatory response to restore tissue homeostasis [5,35]. Continuous imbalance in macrophage functions may be pathogenic and lead to chronic inflammatory and autoimmune diseases as well as fibrosis [13,36]. Atherosclerosis is an example of a chronic inflammatory disease, in which monocytes accumulate in the atherosclerotic lesion and generate foam cells by internalizing lipoproteins which amplify the inflammatory environment and promote fibrosis. Furthermore, apoptosis and necrosis of macrophages contribute to the necrotic core formation of the atheroma, which facilitates thrombosis as a consequence of fibrous cap rupture [37,38]. Similarly, macrophages also play a critical role in many autoimmune diseases characterized by a chronic inflammation such as in rheumatoid arthritis, inflammatory bowel disease and multiple sclerosis. In rheumatoid arthritis, macrophages accumulate in the synovial tissue of the inflamed joints causing synovitis and synovial hyperplasia [39,40]. CD11c^high^ immature macrophages accumulate in the intestinal tract of inflammatory bowel disease patients and the macrophages are characterized by impaired bacterial clearance and excessive cytokine secretion, such as TNF-α and IL-23, which facilitate pathogenic Th17 responses [41]. In multiple sclerosis, macrophages infiltrate the central nervous system promoting an inflammatory environment which induces tissue damage [42,43]. Fibrosis is attained when imbalance of extracellular matrix homeostasis is sustained which adversely affects the function of the tissue [35,44].

Inflammation plays a critical role in development and progression of many cancers [45,46]. In the tumor microenvironment, specialized tumor-associated macrophages (TAMs) supports tumor initiation, progression and metastasis by promoting angiogenesis, immunosuppression and activation of the tumor cells [13,36,47,48,49,50]. This subset of macrophages often possess an anti-inflammatory phenotype and are in many studies identified using CD68, CD163, CD204 and/or CD206 as biomarkers [51]. Further, some TAMs also express multi drug resistance protein 1 supporting chemoresistance as demonstrated on CD163^+^CD204^+^ TAMs in epithelial ovarian cancer [52]. However, the TAMs are not a homogenous subset of cells and they also encompass tumor-suppressive macrophages that instead prevent tumor growth and progression [49,50]. Hence, very low levels of TAMs in the tumor microenvironment seems unfavorable in terms of a worse prognosis compared to intermediate levels as demonstrated in classical Hodgkin lymphomas [53].

## 4. Targeting Macrophages in Inflammatory and Malignant Diseases

**Figure 1:**
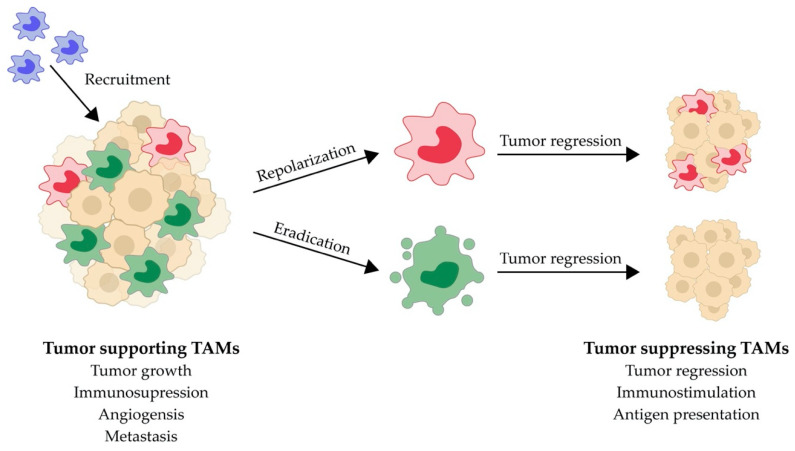
Targeting strategies of tumor associated macrophages (TAMs) in cancer therapy. Some of the tumor recruited macrophages adopt a tumor-supportive phenotype (green) in the tumor microenvironment which is immunosuppressive and supports tumor growth, angiogenesis and metastasis. Anti-tumor effects can be obtained by manipulating the TAM population. First, targeting of monocyte (blue) recruitment to limit TAM density in the tumor. Second, repolarization of tumor-promoting TAMs to tumor-suppressive macrophages (red) may promote tumor regression by stimulating the immune system. Or third, eradication of TAMs may promote tumor regression, either through general TAM depletion or, preferably, through selective depletion of tumor-supportive TAMs.

Over the last decade, modulating macrophage activity as a part of pharmacological therapy of inflammatory and malignant diseases has received increasing interest, leading to the development of multiple drug candidates undergoing clinical trials. Some have even gained clinical approval, as reviewed elsewhere [51,54,55,56,57]. In general, either direct targeting of macrophage receptors or indirect targeting of cytokines secreted by or intended for macrophages are used in macrophage-directed therapy [54]. Targeting macrophages in inflammation is relevant, since the macrophage is the main producer of a range of pro-inflammatory cytokines [58]. Often these cytokines are the direct targets for neutralizing biologics (Infliximab, etanercept, adalimumab, tocilizumab etc.) [59], however targeting of macrophages to lower the cytokine production has also been investigated although without entering clinical development [60]. As the inflammatory state of the tumor microenvironment influence tumor progression, the development of macrophage targeting has led to numerous drug candidates on the market or in clinical trials within anti-cancer therapy. These drugs, suppressing tumor progression and/or metastasis, either block monocyte infiltration (e.g., inhibition of CCL2/CCR2 chemokine axis), repolarize TAMs (e.g., the blocking of CD47 or MARCO or stimulating CD40 or TLRs) or deplete TAMs (e.g., CSF-1R blockade or bisphosphonate toxicity) [51,54,55,56,57] (Figure 1).

To improve target precision specialized drug delivery systems may be used to reduce off-target effects. Antibody-drug conjugates (ADC) represent one promising and popular drug delivery system, which utilizes antibody-specificity to direct small molecules directly to the target of interest. Although only eight ADCs have gained clinical approval so far, at least seventy ADCs are at present in clinical trials [61]. Anti-cancer therapy delivering cytotoxic molecules is the main focus of current ADCs in clinical trials or on the market but the use of the ADC technology for delivery of immunomodulatory molecules in macrophages in inflammatory diseases is also evolving. 

The identification of a surface molecule as target is a prerequisite for efficient drug targeting using ADCs. Ideally, such a target is specific for the target cell in the relevant disease, expressed in relatively high amounts and mediates internalization. The present review describes the use of endocytic receptor CD163 in macrophages as target [62].

## 5. The CD163^+^ Macrophages

The transmembrane scavenger receptor CD163 is expressed exclusively in monocytes (low expression) and macrophages (high expression) [63]. Anti-inflammatory cytokines such as IL-6 and IL-10 induce the expression of CD163 while inflammatory stimuli by IL-4, TNF-α, IFN-γ and LPS repress the expression [64]. Further, LPS has been shown to activate ADAM17 which mediates shedding of CD163 from the cell surface forming soluble CD163 (sCD163) present in plasma and other tissue fluids [65]. The CD163^+^ macrophage population has been associated with anti-inflammatory functions owing to stimulated expression by anti-inflammatory cytokines and its ability to produce anti-inflammatory heme metabolites after CD163-mediated hemoglobin scavenging [63,64]. Further, the anti-inflammatory response to collagen-induced arthritis is hampered in CD163 deficient mice compared to CD163 expressing mice indicating a pivotal role of CD163 in limiting arthritis progression and regression [66]. Additionally, CD163 has also been reported to bind and degrade the inflammatory cytokine tumor necrosis factor-like weak inducer of apoptosis (TWEAK) [67] as well as to recognize and mediate a local immune response to bacteria [68] and internalize virus [69]. CD163 is often used as a M2 marker although it seems apparent that only a subpopulation of M2s are CD163^+^, so in essence a distinct CD163^+^ subpopulation may be defined [70].

CD163 expression is upregulated in a number of diseases although our knowledge of the pathological role of the receptor in disease seems incomplete. Table 1 present a list of inflammatory diseases with up-regulation of CD163-expressing macrophages at the site of inflammation and/or sCD163 in humane fluids. In carotid atherosclerotic plaques, the level of CD163^+^ macrophages correlates with plaque progression and causes a higher risk of myocardial infarction and coronary heart disease [71]. In lupus nephritis, CD163^+^ macrophage infiltration is associated with impaired renal function and correlates with the activity index [72,73]. CD163 expression is elevated in active multiple sclerosis lesions by myelin-laden and perivascular macrophages [74,75]. Further expression is significantly higher in peripheral blood mononuclear cells (PBMC) of relapsing-remitting multiple sclerosis compared to secondary progressive multiple sclerosis demonstrating a phenotypic change during disease progression [76]. In children with non-alcoholic steatohepatitis (NASH), the CD163 level is significantly elevated [77]. However, the expression of CD163 seems unchanged in liver sinusoid between adults with and without NASH [78]. During progression of inflammation, the number of CD163^+^ macrophages increases, this is likely owing to both phenotype changes of local macrophages and macrophage maturation of recruited monocytes. For instance, the amount of CD163^+^ macrophages increases from the acute phase of cutaneous arteritis to the subacute, reparative and healed phase, with highest levels at the subacute phase [79].

sCD163 is a marker of macrophage activation [65] and it has been associated with a number of inflammatory diseases such as atherosclerosis [80,81], hemophagocytic lymphohistiocytosis [82,83,84] and diabetes mellitus [85,86] including diabetic polyneuropathy [87], diabetic ketoacidosis [88] and proliferative diabetic retinopathy [89,90]. Further, a recent paper reviewed sCD163 as a potential biomarker of acute and chronic liver disease and found it elevated in relation to severity in multiple studies [91]. Long-term physical activity also increases sCD163 levels which may be owing to a counteracting effect of CD163^+^ macrophages on exercise-induced pro-inflammatory effects [92]. sCD163 has also been used as a biomarker in other tissue fluids than serum/plasma. For instance, in rheumatoid arthritis and spondyloarthropathy the sCD163 level is elevated in the synovial fluid, where it is associated with disease activity and progression [93,94]. Furthermore, elevated sCD163 levels in sputum, urine, cerebrospinal fluid and vitreous fluid have been shown to be associated with disease activity and treatment in asthma [95,96], lupus nephritis [97,98], multiple sclerosis [99] and proliferative diabetic retinopathy [89,90], respectively.

Generally, CD163 has been used to identify TAMs in malignant diseases and the level of CD163 expressing TAMs has been linked to poor prognosis, overall survival and metastasis of a range of malignancies, as listed in Table 2. Although not known, it is tempting to speculate that a strong anti-inflammatory response in the microenvironment of aggressive tumors contributes to this relationship. Further, the intriguing angiogenic role of CD163^+^ macrophages on the vascular system, as investigated in vessels with atherosclerotic plagues [71] where the macrophages, despite the different pathology compared to cancer, may exert many similar functions to stimulate oxygenation and nutrition of the tissues during the inflammatory process which might contribute to poor prognosis and metastasis. For instance, in gastric cancer CD163^+^ TAMs are significantly correlated with increased microvessel density and poor overall survival [185].

By comparing 13 human malignancies, Jung et al. [186] demonstrated the highest levels of CD163^+^ TAM’s and shortest five-year relative survival rates in pancreas, lung and gallbladder cancers. The expression of CD163 is not only confined to TAMs but also some malignant cells express it as a consequence of cell fusion [187,188,189,190]. This leads to a more invasive and metastatic phenotype causing a worse prognosis as demonstrated in bladder cancer [190], breast cancer [191,192,193] and colorectal cancer [194,195] and so forth. Therefore, it is important to distinguish CD163^+^ malignant cells and macrophages when investigating the influence of CD163 as a measure of tumor microenvironment and its influence on prognosis. This might have influenced some of the results assessed in Table 2.

Despite the overall clear correlation between CD163 expression and poor survival across a range of malignancies, conflicting data has been reported regarding correlation between CD163 expression and survival in multiple malignant diseases. This discrepancy may be attributed to varying quantitative methods of TAMs [294]. Most often CD163^+^ TAMs are measured semi-quantitatively by immunohistochemical staining of CD163 alone or both CD68 and CD163. However, using just one or two markers have been shown to be insufficient to characterize TAMs. For instance, across lymphomas TAMs differ and multiple markers such as S100A9, CCR2, CD36, Slan or CD32 should accompany TAM identification [295]. Further, CD68 and CD163 antibodies must be chosen with caution as different antibodies influence the staining significantly. For instance, anti-CD68 antibody KP-1 stains both macrophages and neutrophils in human non–small cell lung cancer tissue, while anti-CD68 antibody PG-M1 does not [296]. In addition, some anti-CD163 antibodies are dependent on epitope accessibility and extracellular calcium which results in discrepancies among reported levels of CD163. Clone GHI/61 does not recognize CD163 in presence of calcium while RM3/1 only bind CD163 in the presence of calcium, which makes the choice of anticoagulant critical [297]. Finally, cell fusion of macrophages and cancer cells might skew CD163 quantification, as mentioned above.

One example of conflicting data challenges the association between CD163 expression and the overall survival of hepatocellular carcinoma (HCC). The high abundance of CD163-expressing macrophages was found to be associated with a poor prognosis of HCC [258]. However, sCD163 was not associated with overall survival in one study [298] but found as a prognostic factor for overall survival in another study [257]. Yet, sCD163 is not suitable for diagnosis of HCC as it is not able to differentiate patients suffering from HCC and cirrhosis [257]. Kong et al. [299] argue that the upregulation of CD163 and sCD163 is associated with active hepatitis rather than tumor progression. However, a recent study outlines the landscape of immune cells in HCC by single-cell RNA sequencing and revealed upregulation of CD163^+^ TAM in the tumor core and edge [259] supporting the association of HCC, CD163 and prognosis. It is yet unknown if CD163 has a function in disease development per se or the expression level just reflects the inflammatory state of the macrophages in the tumor.

## 6. Targeting CD163^+^ Macrophages

**Figure 2:**
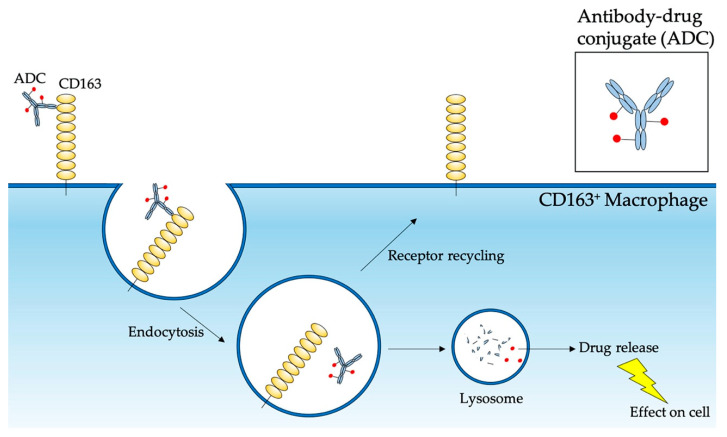
CD163 targeting using antibody-drug conjugates (ADC) in inflammatory and malignant diseases. CD163 is highly expressed on the surface of macrophages in many inflammatory diseases. A CD163-specific ADC is composed of a monoclonal anti-CD163 antibody which is conjugated to an anti-inflammatory or anti-tumor pharmaceutical ingredient by a chemical linker. The ADC binds to CD163 on the macrophage surface which triggers endocytosis of the conjugate. Within the exosome the ADC is released from CD163 and upon lysosome fusion the active pharmaceutical ingredient is released from the antibody to exert its function intracellularly.

The upregulation of expression in a number of inflammatory and malignant diseases makes CD163 a promising target in specific drug delivery to macrophages. Anti-CD163 immunoglobins have been used to direct active pharmaceutical ingredients into CD163^+^ macrophages using ADCs and immunoliposomes to improve efficacy and reduce toxicity (Figure 2) [62]. CD163 is an ideal target for immuno-based therapy as its expression is highly selective for the monocytic linage, expressed in the plasma membrane, endocytoses the ligand within minutes and recycles the receptor to the cell surface [300,301].

Rapid internalization of CD163 binding antibodies has been demonstrated in vivo. In pigs, the plasma half-life of a humanized CD163 monoclonal antibody was in the range of just 5–8 min [302]. Administration of ^68^Ga radiolabeled anti-CD163 antibody in rats revealed biodistribution of the trace 15 min post-administration to the liver, spleen and bone marrow using positron emission tomography (PET) bioimaging [303]. This is in accordance with the high CD163 expression level found in hepatic Kupffer cells, splenic red pulp macrophages and bone marrow macrophages in rats [304]. The half-life of an anti-CD163 monoclonal antibody injected at a dose of 2.4 mg/kg was 20 min in rats. However, injecting 0.05 mg/kg of ^125^I-labeled anti-CD163 monoclonal antibody showed a half-life of just 4 min. The differences in clearance time indicate CD163 targeting is saturable [305]. The half-life reflects the high endocytic capacity of the CD163 system and is in line with the half-life of hemoglobin and haptoglobin-hemoglobin complex after iv injections [306].

Antibody-drug conjugates combine the specificity of monoclonal antibodies for their target antigen with the pharmaceutical activity of the drug by a chemical linker to obtain a selective drug delivery system with limited off-target effects [301]. Conjugation of dexamethasone-hemisuccinate-NHS to anti-CD163 monoclonal antibody did not alter the selectivity nor internalization properties of an anti-CD163 antibody. In vitro, the ADC was internalized and anti-CD163 antibody and dexamethasone were colocalized intracellularly within 30 min. Further within two hours, dexamethasone separated from the conjugate was intracellularly detected by confocal fluorescence microscopy of CD163-transfected CHO cells and rat spleen cells in suspension [305].

Targeting of drugs to CD163^+^ macrophages has also been performed by binding anti-CD163 monoclonal antibodies to the surface of liposomes. Etzerodt et al. incorporated the antibody on the liposome surface using active polyethylene glycol (PEG) and demonstrated selective uptake and degradation of calcein-loaded CD163-conjugated liposomes in human macrophage colony-stimulating factor (M-CSF)-stimulated cultured monocytes by the endocytic pathway [307]. Pegylated liposomes are designated ‘stealth liposomes’ because they possess prolonged therapeutic half-life as they evade the reticuloendothelial system. They have also been claimed to passively accumulate in tumor tissue over healthy tissue due to the enhanced permeability and retention effect of tumor vasculature and thereby minimize toxic side effects in other organs [308,309]. Therefore, stealth liposomes seem advantageous in cancer therapy. However, the first immunoliposome has yet to become approved by the American Food and Drug Administration (FDA) and the European Medicines Agency (EMA) and only few of clinical trials are reported. Limited clinical translation has been proposed to be due to limited tissue distribution and structural instability [310,311].

Both ADC’s and pegylated stealth liposomes have been targeted towards CD163^+^ macrophages using anti-CD163 immunoglobins in in vivo animal models of inflammatory and malignant diseases. Furthermore, anti-CD163 antibodies have been demonstrated as potential traces for bioimaging of CD163^+^ macrophages in diagnosis and progression of diseases.

### 6.1. CD163 Targeting in Inflammation

**Figure 3:**
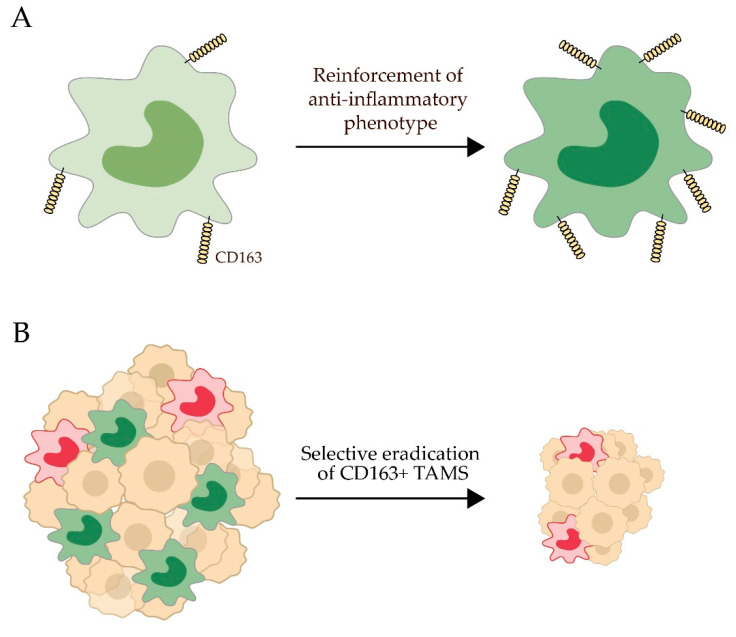
Selective targeting of CD163^+^ macrophages in inflammatory and cancer therapy. (**A**) The anti-inflammatory effects of CD163^+^ macrophages are reinforced by either high dose free dexamethasone or low dose CD163 targeted dexamethasone delivery system. High dose free dexamethasone carries a number of systemic side effects, including lymphocyte apoptosis that can be avoided by low dose dexamethasone targeting. (**B**) Selective eradication of CD163^+^ macrophages entail suppressed tumor growth and reduce metastatic spread in animal models.

Delivery of anti-inflammatory glucocorticoid drugs directly to CD163^+^ macrophages has been demonstrated in different animal models. Glucocorticoids are potent anti-inflammatory drug used to treat inflammatory, autoimmune and endocrine diseases which exert their anti-inflammatory effects on macrophages by influencing their phenotype thereby modulation the expression of cytokines [312]. However, systemic administration of glucocorticoids is associated with a range of dose-dependent side effects including metabolic, endocrine and immunosuppressive effects due to the presence of the glucocorticoid receptor in virtually all cells [312,313]. Targeting dexamethasone—a synthetic glucocorticoid without mineralocorticoid activity—to CD163^+^ macrophages as an ADC has shown promising anti-inflammatory effect in vivo in rats and pigs. Due to the lower needed dose for obtaining pharmacological effect, it has been possible to escape the systemic side effects of free dexamethasone [302,305,314]. The potency of dexamethasone conjugated to an anti-CD163 antibody was shown to be 50-fold higher compared to non-conjugated dexamethasone in in vivo endotoxemia models in both rat and pig [302,305]. The conjugate suppresses the hepatic acute phase response upon LPS treatment significantly, with a 0.02 mg/kg dose of conjugate comparing to the effect of a high-dose (1 mg/kg) free dexamethasone in rats [314]. More importantly, the serious systemic side effects of free dexamethasone (e.g., overall body weight loss, suppressed endogenous cortisol production and lymphocyte apoptosis measured as reduced thymus and spleen weight) were not observed when using the anti-CD163 conjugate at an equipotent dose [302,305,314]. Thus, the antibody-mediated CD163-targeting of dexamethasone is a low dose glucocorticoid therapy with high dose effects on macrophages and thereby a less harmful approach in inflammatory therapy (Figure 3A).

Targeting dexamethasone to CD163^+^ macrophages has shown to be effective in limiting NASH progression [315] and limiting 6-hydroxydopamine (6-OHDA)-induced Parkinson’s disease [316] in rats. NASH is characterized by hepatic steatosis, hepatocyte ballooning and inflammation and is diagnosed by histological evaluation of liver biopsy [317]. In rats fed a high fructose diet for 12 weeks the overall non-alcoholic fatty liver disease activity score was significantly improved in anti-CD163-dexamethasone ADC treated rats compared to controls. The conjugate not only affected inflammation but also reduced hepatic fibrosis significantly, demonstrating the role of the macrophages as an important codriver of hepatic fibrosis.

Neuroinflammation is essential in the pathogenesis of neurodegenerative diseases [318] and myeloid cells are involved in these pathological conditions, for instance in Parkinson’s disease [319]. Targeting dexamethasone to CD163^+^ macrophages entails a neuroprotective effect in the substantia nigra in the rat 6-OHDA-induced disease model of Parkinson’s disease [316]. Infiltration of CD163^+^ macrophages were observed in the striatal 6-OHDA lesions and by using anti-CD163-linked stealth liposomes loaded with dexamethasone, Tentillier et al. [316] demonstrated that the liposomes accumulated in the brain after peripheral administration and that the cargo was delivered to CD11b^+^ macrophages in the brain parenchyma. Repeated administration of CD163-targeted dexamethasone loaded liposomes improved motor performance significantly compared to free dexamethasone and the low dose of the CD163-targeted liposomes did not lead to systemic side effects, which was observed for high-dose free dexamethasone.

During liver resection, Pringle’s maneuver is performed to limit excessive perioperative blood loss, however, this causes cell death and inflammation of the liver tissue due to hypoxia. Glucocorticoids have been shown effective as prophylactic treatment limiting the injuries from ischemia. In a study by Møller et al. [320], prophylactic low dose anti-CD163-dexamathasone ADC significantly reduced the number of apoptotic cells following reperfusion after Pringle’s maneuver in rats. The same effect was accomplished by high-dose unconjugated dexamethasone. However, alanine aminotransferase and alkaline phosphatase levels were significantly elevated in the high dexamethasone treated animals compared to controls which the authors explain as a result of systemic side effects of glucocorticoids. The inflammation in consequence of hepatic resection also slows down the subsequent hepatic regeneration. However, prophylactic treatment with anti-CD163 stealth liposomes loaded with dexamethasone prior to 70% hepatectomy in rats reduced the inflammatory response although it did not influence hepatocyte regeneration rate [321]. 

As an alternative to target dexamethasone to macrophages, Rafique et al. [322] strengthened the anti-inflammatory effect of another steroid, calcitriol (vitamin D) on human monocytes in vitro using CD163-targeted pegylated nanoparticles loaded with calcitriol. M-CSF/ Granulocyte-macrophage colony-stimulating factor (GM-CSF) differentiated human macrophages from buffy coats were incubated with either non-targeted stealth calcitriol liposomes or stealth liposomes with CD163-antibody or control IgG for 24 h before induction of inflammation by LPS for 4 h. The CD163-targeted calcitriol treatment reduced the mRNA expression of anti-inflammatory markers and increased the expression of the pro-inflammatory cytokine, IL-10. However, a comparable anti-inflammatory effect was observed when treating the cells with IgG-targeted and non-targeted liposomes which was explained as internalization upon Fc receptor binding or phagocytosis, respectively. Therefore, the anti-inflammatory effects may not solely be attributed to the targeting of CD163. Distribution of the liposomes in vivo was investigated using Xenogen in vivo imaging system (IVIS), which revealed accumulation of CD163-targeted liposomes in the liver within 15 min while non-targeted liposomes circulated in the blood stream for more than three and a half hours [322]. This might indicate that CD163-targeting of calcitriol is favorable and specific as Kupffer cells express CD163. However, in vivo experiments are needed to elucidate the anti-inflammatory effect of targeting calcitriol to macrophages and the effect on other cell types needs to be ruled out.

The CD163-targeted nanoparticles have also been used for optimization of diagnostic procedures. CD163 may act as a potential marker of activated macrophages in pathological conditions and be utilized to determine disease status and progression using noninvasive magnetic resonance imaging (MRI) [126,323] or positron emission tomography (PET) [303].

Conjugating anti-CD163 antibody to gold-coated iron oxide have been found advantageous in specific MRI detection of CD163^+^ macrophages in atherosclerotic lesions [323] and rhabdomyolysis-induced acute kidney injury [126] in mice models. In vitro, the targeted nanoparticles reduced T2 values in CD163-expressing human monocyte-derived macrophages (PMA-differentiated THP-1 cells treated with dexamethasone) and murine peritoneal macrophages (treated with dexamethasone). Pretreatment with anti-CD163 antibody before adding the nanoparticles neutralized the effect indicating the significance of CD163 targeting [323]. The signal intensity of MRI also decreased significantly in vivo after injection of CD163-targeted gold-coated iron oxide in both animal models, indicating accumulation of CD163^+^ macrophages in the lesioned aorta and kidneys [126,323].

PET imaging of rats with collagen-induced arthritic after ^68^Ga radiolabeled anti-CD163 antibody administration revealed significant accumulation of CD163^+^ macrophages in the rear-inflamed paws compared to healthy rats. However, the overall accumulation of ^68^Ga-labeled anti-CD163 was low in the paws compared to the liver and spleen of both healthy and arthritic rats [303].

### 6.2. CD163 Targeting in Malignant Disaeses

CD163 expression on TAMs correlates with poor prognosis in a number of human malignant tumors (Table 2) [49,62,324]. Therefore, specific targeting of CD163^+^ macrophages may contribute to current cancer therapy. Doxorubicin-loaded stealth liposomes coated with an anti-CD163 monoclonal antibody [307] have shown promising results regarding tumor regression in mouse models of PD-1 therapy resistant melanomas [325] and metastatic ovarian cancer [326]. CD163^+^ TAMs represent a pro-tumorigenic fraction of TAMs that possess immunosuppressive characteristics in the melanoma mouse model. Specific depletion of this subset of TAMs by the anti-CD163 lipid nanoparticles containing doxorubicin resulted in reduced tumor growth and increased infiltration of monocytes and immature TAMs that advance inflammatory responses and recruitment of CD4^+^ and CD8^+^ T cells promoting tumor regression. Compared to anti-CSF-1R blocking antibody causing general TAM depletion, specific depletion of CD163^+^ TAMs was shown to be a more efficient inhibitor of tumor growth [325]. In the mouse model of metastatic ovarian cancer, tissue-resident CD163^+^, Tim4^+^ macrophages in the omentum (the fat deposit in the peritoneal cavity) contributed significantly to the metastatic spread of ovarian cancer cells and the development of invasive disease. Depletion of both monocyte derived CD163^+^ macrophages and tissue resident CD163^+^ Tim4^+^ macrophages by CD163-targeted lipid nanoparticles loaded with doxorubicin contributed to reduced tumor growth in omentum and reduced metastatic spread in the ascites and to the diaphragm [326] (Figure 3B).

As a less radical alternative to eradication of CD163^+^ TAMs, the macrophages might be reprogrammed from a tumor-promoting “M2-like” phenotype to a tumoricidal “M1-like” phenotype. This has been demonstrated in vitro by Andersen et al. using pegylated liposomes loaded with corosolic acid (vitamin D) [327]. Corosolic acid inhibits STAT3, which is an oncogene increased in several human malignancies [328] and an important regulator of CD163 in gastric cancer cells [252]. CD163 antibody mediated specific targeting of IL-10 stimulated CD163^+^ monocyte-derived human macrophages with corosolic acid produced significant reduced gene expression of IL-10 and CD163 and significant induced gene expression of INF-γ, TNF-α, IL-2 and IL-12 [327]. However, in vivo experiments are necessary to fully evaluate the effect of the specific targeting of STAT3 inhibitors to TAMs. It will be interesting to see whether the specific direction of the STAT3 inhibitor to tumor-promoting macrophages reduces the risk of autoimmune side effects of STAT3 inhibition as previously reported [329,330].

So far, only immunoliposomes targeting CD163 have been investigated in animal models of cancers. No immunoliposomes have gained clinical approval [310,311] whereas multiple ADCs have clinical approval though or have entered clinical trials [61]. In a drug-development perspective, it would therefore be highly relevant also to investigate CD163 targeting ADCs as a drug delivery system in cancer therapy.

## 7. Concluding Remarks

Macrophages are heterogenic and plastic immune cells that take part in numerous vital functions throughout the body. Their plasticity has previously been described as a polarized dichotomy of pro-inflammatory M1 macrophages or anti-inflammatory M2 macrophages. However, this nomenclature is inadequate as a spectrum of macrophages exists with intermediate phenotypes changing over time.

Persistent imbalance in the macrophage population contribute to pathogenesis of a range of inflammatory, autoimmune and malignant diseases. Therefore, targeting of macrophages in anti-inflammatory and anti-cancer therapy can contribute to treatment of these diseases avoiding off-target effects. The macrophage scavenger receptor CD163, which is upregulated in a number of inflammatory and malignant diseases, is a promising target for such delivery. Dexamethasone-conjugated anti-CD163 ADCs have especially shown promising results in simple endotoxemia and inflammatory disease models in rodents and pigs. Similar anti-inflammatory effects of dexamethasone were obtained using a 50-fold lower concentration when comparing CD163 targeted dexamethasone with free dexamethasone. Furthermore, the ADC technology evaded the serious side effects of dexamethasone, in essence making it a low dose glucocorticoid therapy systemically but with local high dose efficacy on macrophages. Depletion of CD163^+^ TAMs using anti-CD163 immunoliposomes loaded with doxorubicin limits tumor progression in malignant animal models. However, the limited approval of immunoliposomes as a drug delivery system suggests that ADCs are more reliable in CD163 targeting although more evidence on efficacy and toxicity in animal disease models is warranted before entering clinical trials. 

In conclusion, the specific targeting of CD163^+^ macrophages has been demonstrated to be a promising drug delivery strategy for handling inflammatory and malignant disease, contributing to the current pharmaceutical therapies.

## Figures and Tables

**Table 1:** Enhanced expression of CD163+ macrophages at site of inflammation and increased soluble CD163 (sCD163) in human inflammatory diseases.

Disease	CD163 ^1^	sCD163 ^1^	References
**Infectious Inflammations**			
Sepsis	↑	↑	[100,101,102]
HIV	↑	↑	[103,104,105,106]
Acute viral hepatitis	↑		[107]
Chronic viral hepatitis	↑	↑	[108,109]
Malaria	↑ ^2^	↑	[110,111,112,113]
**Acute inflammations**			
Hemophagocytic lymphohistiocytosis	↑	↑	[82,83,84,114]
Acute Coronary Syndromes	↑	↑	[115,116]
Peripheral artery disease		↑	[117]
Acute-on-chronic liver failure	↑ ^2^	↑	[91,118,119,120]
Acute liver failure		↑	[121]
Alcoholic hepatitis	↑	↑	[122,123]
Acute kidney injury	↑	↑	[124,125,126,127,128]
Kidney allograft rejection	↑		[129,130,131]
Acute graft-versus-host disease	↑		[132]
**Chronic inflammations**			
Atherosclerosis	↑	↑	[71,80,81,133,134,135]
Atrial fibrillation	↑	↑	[136,137]
Chronic heart failure		↑	[138,139,140]
Chronic graft-versus-host disease	↑		[141,142]
Sickle cell disease		↑	[143]
Cirrhosis		↑	[144,145]
Non-alcoholic steatohepatitis	↑/→	↑	[77,78,146,147,148]
Type 1 diabetes mellitus		↑	[85,88]
Type 2 diabetes mellitus	↓ ^2^	↑	[86,87,149,150,151,152]
Proliferative Diabetic Retinopathy	↑	↑	[89,90]
Gestational diabetes mellitus	↑	↑	[153,154,155]
Crohn’s disease	↑	↑	[156,157,158]
Ulcerative colitis	↑	↑	[156,158,159]
Celiac disease		↑	[160]
Asthma	↑	↑	[95,96,161]
Sarcoidosis	↑	↑	[162,163,164,165,166]
Glomerulonephritis	↑		[72]
Lupus nephritis	↑	↑	[72,73,97,98,167]
Systemic lupus erythematosus	↑ ^2^	↑	[135,168,169]
Rheumatoid arthritis	↑	↑	[93,94,170,171]
Spondyloarthropathy	↑	↑	[94,157,172,173]
Sjögren’s Syndrome	↑		[174,175]
Osteoarthritis	↑	↑	[176,177]
Scleroderma	↑	↑	[23,178,179,180,181]
Multiple sclerosis	↑	↑	[74,75,76,99,182,183]
Alzheimer’s disease	↑		[184]
Parkinson’s disease	↑		[184]

^1^ Increased (↑), decreased (↓) or unchanged (→) expression of CD163/sCD163 compared to controls; ^2^ Demonstrated in peripheral blood mononuclear cell (PBMC).

**Table 2:** Malignant diseases with proven correlation between CD163^+^ tumor-associated macrophages and reduced survival.

Malignancy	References
Classic Hodgkin lymphoma	[196,197,198,199,200,201]
Diffuse large B-cell lymphoma	[202,203,204,205,206]
T-cell lymphomas	[207,208,209,210]
Multiple myeloma	[211,212,213,214] ^1^
Glioma (incl. Glioblastoma)	[215,216,217,218,219,220,221] ^2^
Embryonal rhabdomyosarcoma	[222]
Non-small Cell Lung Cancer	[223,224,225,226,227,228,229]
Head and neck squamous cell carcinoma	[230,231]
Oral Squamous cell carcinoma	[232,233,234,235,236,237,238,239] ^2^
Nasopharyngeal carcinoma	[240,241]
Laryngeal squamous cell carcinoma	[242,243]
Esophageal squamous cell carcinoma	[244,245,246,247,248,249]
Gastric cancer	[185,250,251,252,253,254] ^1^
Colorectal cancer	[194,195,255,256] ^2^
Hepatocellular carcinoma	[257,258,259] ^1^
pancreatic ductal adenocarcinoma	[260,261,262,263,264,265,266] ^2^
Clear Cell Renal Cell Carcinoma	[267,268] ^2^
Bladder cancer	[190,269,270,271,272]
Ovarian cancer	[273,274,275,276,277] ^1^
Endometrial adenocarcinoma	[278]
Breast cancer	[191,192,193,279,280,281,282,283,284,285] ^2^
Malignant melanomas	[286,287,288,289,290,291,292,293] ^1,2^

^1^ Correlation between soluble CD163 (sCD163) and reduced survival; ^2^ CD163 expressing tumor cells documented in peripheral blood mononuclear cell (PBMC) or solid tumor.

## Abbreviations

6-OHDA	6-hydroxydopamine
ADAM17	A disintegrin and metalloproteinase 17
ADC	Antibody-drug conjugate
CD163	Cluster of differentiation 163
CSF-1R	Colony stimulating factor 1 receptor
CXCL	Chemokine ligand
EMA	European Medicines Agency
EMR1	EGF-like module-containing mucin-like hormone receptor-like 1
FDA	U.S. Food and Drug Administration
GM-CSF	Granulocyte-macrophage colony-stimulating factor
HCC	Hepatocellular carcinoma
IFN-γ	Interferon-gamma
IL	Interleukin
iNOS	Nitric oxide synthase
IVIS	In vivo imaging system
LPS	Lipopolysaccharide
MARCO	Macrophage receptor with collagenous structure
M-CSF	Macrophage colony-stimulating factor
MRI	Magnetic resonance imaging
NASH	Non-alcoholic steatohepatitis
PBMC	Peripheral blood mononuclear cells
PD-1	Programmed cell death protein 1
PEG	Polyethylene glycol
PET	Positron emission tomography
PGE_2_	Prostaglanding E_2_
sCD163	Soluble CD163
STAT3	Signal transducer and activator of transcription 3
TAM	Tumor-associated macrophages
TGF-β	Transforming growth factor beta
TLR	Toll-like receptor
TNF-α	Tumor necrosis factor alpha
TWEAK	Tumor necrosis factor-like weak inducer of apoptosis
